# HPeak: an HMM-based algorithm for defining read-enriched regions in ChIP-Seq data

**DOI:** 10.1186/1471-2105-11-369

**Published:** 2010-07-02

**Authors:** Zhaohui S Qin, Jianjun Yu, Jincheng Shen, Christopher A Maher, Ming Hu, Shanker Kalyana-Sundaram, Jindan Yu, Arul M Chinnaiyan

**Affiliations:** 1 Center for Statistical Genetics, Department of Biostatistics, School of Public Health, University of Michigan, 1415 Washington Heights, Ann Arbor, MI 48109-2029, USA; 2 Center for Computational Medicine and Bioinformatics, University of Michigan Medical School, 100 Washtenaw Avenue, Ann Arbor, MI 48109-2218, USA; 3 Michigan Center for Translational Pathology, University of Michigan Medical School, 1400 E. Medical Center Drive, Ann Arbor, MI 48109-0940, USA; 4 Department of Pathology, University of Michigan Medical School, 1301 Catherine, Ann Arbor, Michigan 48109-0602, USA; 5 Division of Hematology/Oncology, Robert H. Lurie Comprehensive Cancer Center, Northwestern University, 676 North St. Clair, Suite 1200, Chicago, IL 60611, USA; 6 Comprehensive Cancer Center, University of Michigan Medical School, 1500 E. Medical Center Drive, Ann Arbor, MI 48109-0944, USA; 7 Department of Urology, University of Michigan Medical School, 1500 E. Medical Center Drive, Ann Arbor, MI 48109-0330, USA; 8 Howard Hughes Medical Institute, 4000 Jones Bridge Road, Chevy Chase, MD 20815-6789, USA

## Abstract

**Background:**

Protein-DNA interaction constitutes a basic mechanism for the genetic regulation of target gene expression. Deciphering this mechanism has been a daunting task due to the difficulty in characterizing protein-bound DNA on a large scale. A powerful technique has recently emerged that couples chromatin immunoprecipitation (ChIP) with next-generation sequencing, (ChIP-Seq). This technique provides a direct survey of the cistrom of transcription factors and other chromatin-associated proteins. In order to realize the full potential of this technique, increasingly sophisticated statistical algorithms have been developed to analyze the massive amount of data generated by this method.

**Results:**

Here we introduce HPeak, a **H**idden Markov model (HMM)-based **Peak**-finding algorithm for analyzing ChIP-Seq data to identify protein-interacting genomic regions. In contrast to the majority of available ChIP-Seq analysis software packages, HPeak is a model-based approach allowing for rigorous statistical inference. This approach enables HPeak to accurately infer genomic regions enriched with sequence reads by assuming realistic probability distributions, in conjunction with a novel weighting scheme on the sequencing read coverage.

**Conclusions:**

Using biologically relevant data collections, we found that HPeak showed a higher prevalence of the expected transcription factor binding motifs in ChIP-enriched sequences relative to the control sequences when compared to other currently available ChIP-Seq analysis approaches. Additionally, in comparison to the ChIP-chip assay, ChIP-Seq provides higher resolution along with improved sensitivity and specificity of binding site detection. Additional file and the HPeak program are freely available at http://www.sph.umich.edu/csg/qin/HPeak.

## Background

Understanding transcriptional regulation is essential to deciphering the genetic pathways involved in various cellular processes and represents one of the major challenges in molecular biology. One critical step during this process is to determine how proteins interact with target DNA to regulate gene expression. Chromatin immunoprecipitation (ChIP) followed by PCR amplification of specific target DNA has been the primary approach to detect *in vivo *protein-DNA interaction [1,2]. However, the ChIP-PCR assay has been limiting in characterizing ChIP-enriched genomic DNA on a genome scale. To address this, various techniques have been developed to identify the binding sites of specific DNA-associated proteins [3]. One frequently used technique is ChIP-chip [4-6], in which the protein-bound DNA is detected through hybridization to DNA microarrays containing a fixed set of probes. However, this approach is heavily biased towards the predetermined probes selected on the DNA microarray, limiting the scale and resolution of this method.

More recently, ChIP-Seq, leveraging massively parallel next-generation sequencing technology, has emerged as a powerful method for genome-wide mapping of protein-DNA interactions and histone modifications [7-9]. Using this technology, numerous studies have been conducted to characterize the genomic landscape of various transcription factors (TFs), histone marks and methylation patterns [10-19]. In ChIP-Seq experiments, the ChIP process isolates DNA fragments bound by a protein using a corresponding antibody. Oligonucleotide adapters are then linked to the DNA to allow ultra-high-throughput sequencing. Through direct sequencing of all of the ChIP-enriched DNA fragments, ChIP-Seq is capable of revealing protein-DNA interaction sites across the entire genome, making it a valuable tool for researchers.

An array of computer algorithms has been developed to analyze ChIP-Seq data aiming to identify ChIP-enriched regions [10,11,20-31]. Excellent reviews of these methods can be found in Spyrou et al. 2009 [28] and Laajala et al. 2009 [32]. A brief description of the seven methods chosen for comparison in this study can be found in the Method section. Although performed well in ChIP-Seq studies, the majority of these methods are rule-based therefore lack the ability to determine the *significance *of each region. To address this, we have adopted a probability model-based approach to explicitly model noise within sequencing data, thereby enabling rigorous statistical inference. For example, the probability of enrichment can be derived and used to compare across samples and experiments. Our approach, referred to as HPeak, utilizes a hidden Markov model (HMM). HMMs have been successfully applied for the analysis of ChIP-chip data [33-37], which motivated us to adopt HMM in our present algorithm. Recently, Mikkelsen et al. (2007) [12] and Xu et al. (2008) [24] have utilized HMMs in their ChIP-Seq studies. However, very little detail of their HMM is provided in Mikkelsen et al. and the ChIPDiff method presented in Xu et al. is restricted to analyzing comparative histone modification data. By using a novel unbalanced weighting scheme, HPeak will account for the uncertainties in the actual lengths of DNA fragments. Therefore, it is capable of accurately reconstructing the genome-wide coverage profiles of DNA fragments. Such information can be used to define the boundaries of ChIP-enriched regions, which is indicated by the significantly elevated DNA fragment coverage relative to the neighboring genomic regions. Overall, we demonstrated that HPeak produces higher motif enrichment in the peaks identified without sacrificing sensitivity when compared with other existing peak-calling algorithms.

## Results

### Datasets

To demonstrate the performance of the HPeak algorithm, in this study we used four previously published ChIP-Seq data sets including the NRSF (neuronrestrictive silencer factor) dataset [10], the STAT1 (signal transducer and activator of transcription protein 1) dataset [11] and datasets from two histone marks H3K4me3 and H3K27me3 [8]. We selected these two histone mark datasets because both H3K4me3 and H3K27me3 are important histone modifier and they show opposite modification patterns [13].

The NRSF ChIP-Seq data [10] was downloaded from a website of Illumina (Illumina, Inc. San Diego, CA), now accessible from GEO with accession number GSE13047. It consists of two files of 25 bp sequencing reads in ELAND output file format. Only reads that uniquely mapped to the human reference genome, with up to two mismatches, were included in these two files. One file has about 1.7 million reads that were obtained from the sample treated by ChIP. The other file contains about 2.3 million reads obtained from the mock control sample.

The STAT1 dataset [11] was downloaded from http://www.bcgsc.ca/data/chipseq. It consists of two files in ELAND output file format, each of which contains reads combined from six lanes of a flowcell. The lengths of reads contained in these two files range from 21 to 27 bp. Only reads that uniquely mapped to the human reference genome, with up to two mismatches, were included in these two files. The numbers of uniquely-mapped reads contained in these two files are 15.3 and 13.0 million for the stimulated and the unstimulated samples respectively.

The BED format of aligned reads obtained from H3K4me3 and H3K27me3 ChIP-Seq experiments [8] were downloaded from http://dir.nhlbi.nih.gov/papers/lmi/epigenomes/hgtcell.aspx. Reads contained in these two files are 24 bp in length. The numbers of available reads contained in these two files are 16.8 and 9.0 million respectively.

### ChIP-Seq reproducibility

It is of critical importance to examine the reproducibility of an experimental assay to confirm that it returns consistent results on biological and technical replicates. In this study, we evaluated the reproducibility of ChIP-Seq by examining the similarity of genome-wide distributions of sequencing reads obtained from duplicated or distinct samples. A common strategy is to apply a Chi-square test of homogeneity to compare the two distributions; however, it faces the challenge of selecting the quantity and size of intervals to cover the genome. In this study, we took an alternative approach using the two-sample Kolmogorov-Smirnov (K-S) test to evaluate the distribution of sequencing reads across chromosomes. To compare under the same setting, we first separated reads into individual chromosomes and forward/reverse strands. Under the null hypothesis that the two samples are reproducible, the genome-wide distributions of sequencing reads are assumed to be identical. Thus we expect non-significant outcome from the K-S test conducted in each chromosome/strand combination. Bonferroni correction was used to correct for multiple testing.

For our reproducibility analysis we used the STAT1 ChIP-Seq data [11], as this dataset is comprised of reads from multiple lanes. In total, there are six lanes each for the stimulated and unstimulated samples. The variance between the numbers of usable reads differed substantially between lanes, ranging from 0.7 million to 4.3 million reads per lane of stimulated sample, and 0.6 million to 3.4 million per lane of unstimulated sample. Since differences in sequencing depth affect the reproducibility assessment, we only considered pairs of lanes with similar numbers of uniquely mapped reads (within 10%). Table 1 lists the numbers of chromosome/strand combinations in which the p-value from the K-S test is lower than the Bonferroni corrected significance threshold of 0.001. As expected, we found that no more than one out of 48 chromosome/strand combinations showed significant discrepancy for a pair of lanes within either the stimulated or the unstimulated samples. When comparing lanes from the stimulated group to the unstimulated group, we observed a much higher frequency of chromosome/strand combinations displaying significant discrepancies (36 to 42 out of 48). Figure S1 in additional file 1 shows plots of empirical cumulative distribution functions (ECDFs) under various scenarios. These observations led us to conclude that the K-S test, along with the ECDF plots, provide a rigorous quantitative means for assessing reproducibility in the ChIP-Seq assay.

**Table 1:** Summary of reproducibility from two-sample Kolmogorov-Smirnov tests performed on the STAT1 ChIP-Seq data*.

Stimulated					
Lanes	1:2	5:6	7:8		
Significant	0	0	0		

**Unstimulated**					

Lanes	2:5	3:4	7:8		
Significant	1	1	0		

**Stimulated vs unstimulated**					

Lanes	1:3	1:4	2:3	2:4	5:7
Significant	38	40	42	40	36

*numbers displayed in the table are quantities of chromosome/strand combinations that show significant discrepancy when conducting the two-sample Kolmogorov-Smirnov test.

### Comparison of ChIP-Seq with ChIP-chip data

**Figure 1:**
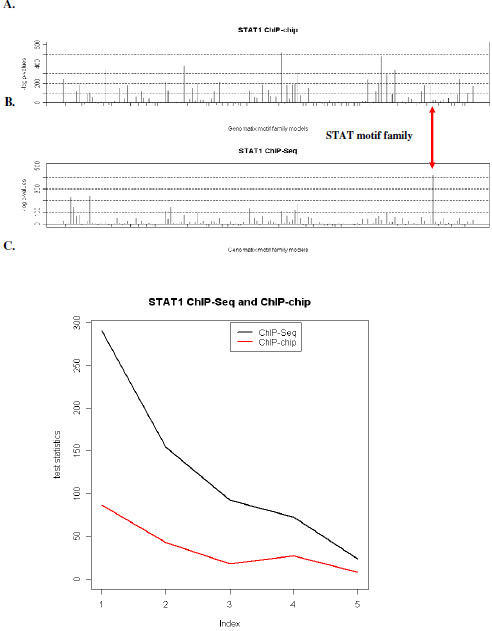
**Comparison of motif enrichment in peaks identified by ChIP-chip and Chip-Seq**. Chi-square test statistics from 2 × 2 contingency table is shown for all 153 families of vertebrate TF binding motif patterns found in the MatBase library 7.0 database of Genomatix (Genomatix, GmBH, Munich, Germany). Motif scan was performed using MatInspector in Genomatix using the default setting. **A**. STAT1 ChIP-chip result (on about 10% of the entire genome, the majority of them (88%) on chromosomes 20, 21, 22, X and Y). **B**. STAT1 ChIP-Seq result (subset of 2,023 peaks out of 24,394 located on Chromsome 20, 21, 22, X and Y). **C**. Correlation between motif enrichment and rank of significance in peaks indentified from STAT1 ChIP-Seq and ChIP-chip experiments. All peaks were ordered according to their significance and then divided into five segments of equal sizes. Their motif enrichment is measured by Chi-square test statistics in these five segments are shown from left to right.

One question that arose from the analysis of the ChIP-Seq experiment is how well it performs relative to the ChIP-chip experiment. We selected STAT1 for a comparison study, since it has been recently evaluated using the ChIP-chip technique [38]. Using a threshold of false discovery rate (FDR) [39] 0.05, Euskirchen et al. identified 3,701 ChIP-enriched regions in about one-tenth of the genome, mostly on chromosomes 20, 21, 22, X and Y. For ease of comparison, we focused only on ChIP-Seq peaks located within these five chromosomes, which corresponds to 2,023 out of 24,394 regions. A motif scan demonstrated that the peaks defined by ChIP-Seq showed greater enrichment of the STAT1 motif than peaks defined by ChIP-chip. Comparison of Chi-square statistics using the ChIP-Seq data showed that motifs in the STAT family are much more enriched relative to motifs from other families (the negative log transformed p-value for STAT family is about twice as large as the second highest) whereas the STAT motif family is not the most enriched in the ChIP-chip data (Figure 1A, B). Our results supported earlier reports of the superior resolution of the ChIP-Seq technique [10].

It has been reported that regions with higher ChIP-chip scores are more likely to contain the motif(s) of interest [40]. A natural question is whether the ChIP-Seq data shows a similar property. To investigate this, we ranked all peaks identified by HPeak from the STAT1 ChIP-Seq data according to the average posterior probabilities of all bins within the peak. We then divided these peaks into five groups of equal sizes. We next calculated the Chi-square test statistics of motif enrichment within each of these five groups of sequences relative to the length-matched control sequences. For comparison, we performed the same analysis on regions identified by the ChIP-chip study on STAT1 [38]. We found that the change in motif enrichment is more dramatic among regions identified by ChIP-Seq than ChIP-chip (Figure 1C).

### Performance comparison with other ChIP-Seq peak-calling algorithms

To demonstrate the performance of HPeak, we tested it on the four publicly available ChIP-Seq datasets described above. Seven ChIP-Seq analysis packages were used in this comparison, PeakFinder [10], FindPeak [11,30] MACS [20], SISSRs [23], CisGenome [22], ChIPseeqer http://physiology.med.cornell.edu/faculty/elemento/lab/chipseq.shtml and ChIPDiff [24] (only used for analyzing histone mark data). We applied these software programs to the datasets using either the default or the recommended parameters according to the program manuals.

Since FindPeaks analyzes treated and control samples separately. To provide a fair comparison, we ran HPeak twice, first using both treated and control samples and second with the treated sample only (comparable to FindPeaks).

**Figure 2:**
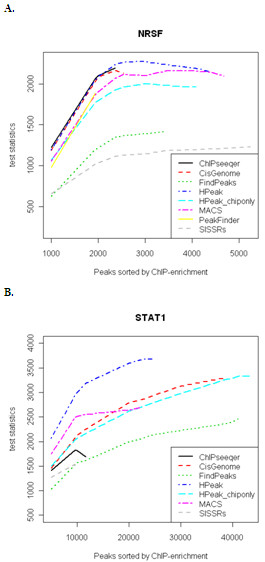
**Performance comparison between HPeak (using data from both treated and untreated samples or using data from treated sample only) and other ChIP-Seq analysis algorithms**. **A**. NRSF ChIP-Seq data: Chi-square test statistics of motif enrichment comparing original sequences under peaks and a set of random control sequences. **B**. STAT1 ChIP-Seq data: Chi-square test statistics of motif enrichment comparing original sequences under peaks and a set of random control sequences.

A brief summary of the peak-calling results of the NRSF ChIP-Seq data is presented in Table 2. The number of identified peaks ranges from 1,935 (Peak Finder) to 5,243 (SISSRs). As explained in the Method section, we used motif enrichment as the measure of performance to evaluate the results obtained from different ChIP-Seq analysis programs. The motif enrichment in the ChIP-enriched and random control sequences was measured by Chi-square test statistics derived from 2 × 2 contingency tables (see the Methods section). Since the numbers of peaks identified differed substantially across these methods, for a fair comparison, we ranked the peaks by each method s specific significance criterion (score or number of reads in peaks), and subsequently selected the top *m *peaks nominated from each approach to compare their motif enrichment (Figure 2A). Overall, for the NRSF dataset, we found HPeak performs the best in most cases when both the stimulated and unstimulated samples were used. When only the stimulated sample was used, HPeak outperformed FindPeaks in terms of enrichment of the expected motif.

**Table 2:** Summary of peaks identified by various peaking calling algorithms.

	Peak Finder^a^	MACS	HPeak^b^	FindPeaks	HPeak (chip only)	ChIPseeqer (control)	SISSRs	CisGenome
NRSF								
chip: 1.7M								
mock: 2.3M								
Number of peaks	1,935	4,679	4,404	3,445	4,085	2,361	5,243	2,545
Covered space (kb)	908	1,902	1,112	4,936	1,512	682	276	775
Avg peak width (bp)	469	406	253	1,433	370	289	53	304

STAT1								
stimulated: 15.3M								
unstimulated: 13.0M								
Number of peaks	-	22,402	24,490	41,127	43,443	11,662	9,561	38,878
Covered space (kb)	-	16,940	6,562	46,781	15,354	3,025	455	10,012
Avg peak width (bp)	-	756	269	1,137	353	259	48	258

H3K4me3								
16.8M								
Number of peaks	28,960	27,568	-	33,890	41,217	31,773	137,286	46,261
Covered space (kb)	30,610	36,675	-	83,348	30,435	18,789	6,464	26,500
Avg peak width (bp)	1,057	1,330	-	2,459	738	591	47	573

H3K27me3								
9.0M								
Number of peaks	335	1,342	-	8,348	4,858	417	2,458	437
Covered space (kb)	83	607	-	19,234	894	115	138	191
Avg peak width (bp)	248	452	-	2304	184	276	56	436

Next, we compared motif enrichment in peaks identified by one method, but not the other, in order to determine the distinguishing features of each program. We found that, among the 2,323 peaks that were identified as significant by HPeak but not PeakFinder, the NRSF motif is the most enriched relative to length-matched random control sequences (Chi-square test p-value < 10^-16^) among all 508 motifs in the MatBase matrix library 7.0. Analogously, among the 495 peaks that were identified by HPeak, but not FindPeaks, the NRSF motif is again the most enriched (Chi-square test p-value 5.8 × 10^-14^). In contrast, among the 285 peaks that were identified by FindPeaks but not HPeak, the NRSF motif ranked 68th among the 508 motifs tested with p-value 0.012, which was no longer significant after correction for multiple testing. When tested using the two half sites of NRSF motif, which have been shown to present in NRSF ChIP-Seq binding domains in statistical significant manner relative to random sequences [10], we found that the right half-site ranked 32^nd ^with p-value of 0.0015 while the left half site ranked 213^th ^with p-value 0.868. Neither was significant after correction for multiple testing. Overall, this suggests that peaks identified by other programs but not by HPeak are likely to be false positives.

We next assessed the performance of HPeak using the STAT1 ChIP-Seq dataset [11]. We did not compare it to PeakFinder, since it only identified approximately one-tenth of the peaks found by most of the other algorithms under its default parameter settings (modifications of the significance threshold failed to result in much increase in the number of peaks). Compared to the NRSF dataset, the STAT1 data represents a deeper coverage scenario, with an increase in usable reads obtained from multiple lanes.

For this dataset, when both stimulated and unstimulated samples were considered under the default setting, HPeak identified 24,394 peaks. The number of peaks identified by competing algorithm ranges from 9,561 (ChIPseeqer) to 41,127 (FindPeaks). Using both the stimulated and unstimulated samples, HPeak showed the highest motif enrichment. When only the stimulated sample was used, HPeak identified 43,440 peaks. Using the stimulated sample alone, HPeak showed significantly higher motif enrichment than FindPeaks. However, adding unstimulated control samples improved HPeak's performance in terms of motif enrichment (Figure 2B). This suggests that the use of control samples may improve the accuracy of identified peak regions as substantial region-specific biases are often observed in genome-wide sequencing due to the different accessibility of genomic regions.

In the above comparison, the lengths of the peaks differed across tested algorithms. Therefore, if we scan these peaks only, those with wider peaks will contain more motif of interest. We have corrected such bias by introducing size-matched control sequences. An alternative solution to remove the size bias is to search a fixed window around the peak summit. This method was adopted in the MACS study [20]. We have examined the performance using this method as well. The results of these scans can be found in Figure S2 in additional file 1. From there, we found that nearly all methods reported similar number of motif occurrences in the 200 bp regions around the peak summits especially for the NRSF data. MACS showed slight edge perhaps owing to its accurate positioning of the peak summits.

For the two histone mark datasets, since there is no list of gold standard binding loci, and there are no known motifs associated with either mark, we tabulated the sets of peaks identified from all peak-calling algorithms and studied the overlap among these sets of peaks. The results are summarized in Tables 2 and 3. We found that results from most of the peak-calling algorithms we tested are very similar especially for the open chromatin histone mark H3K4me3 (most overlap percentages are greater than 90%). For H3K4me3, the overlap pattern is similar to what we observed for TFs NRSF and STAT1 (Table S1 in additional file 1). For H3K27me3, the percentages of overlap vary greatly, most likely due to the fact that the numbers of peaks generated by these peak-calling algorithms are quite different. We did not included results from ChIPDiff in this evaluation since this program produces many more peaks than others and ChIPDiff was originally designed for the comparison between two cell types.

**Table 3:** Summary of overlaps among peaks identified by different peaking calling algorithms in H3K4me3 and H3K27me3 ChIP-Seq datasets*.

H3K4me3							
**Method (# of peaks)**	**Peak Finder**	**MACS**	**HPeak**	**Find Peaks**	**ChIP seeqer**	**SISSRs**	**CisGenome**

Peak Finder (28,960)		84.3	99.8	100.0	98.2	98.0	99.6
MACS (27,568)			90.6	95.4	84.8	87.7	84.8
HPeak (41,217)				99.4	100	98.9	97.1
FindPeaks (33,886)					100	99.8	100
ChIPseeqer (31,773)						99.0	100
SISSRs (137,286)							94.6
CisGenome (46,261)							

**H3K27me3**							

**Method (# of peaks)**	**Peak Finder**	**MACS**	**HPeak**	**Find Peaks**	**ChIP seeqer**	**SISSRs**	**CisGenome**

Peak Finder (335)		44.8	99.7	100	77.3	90.4	70.9
MACS (1,341)			42.5	42.4	23.5	24.4	32.9
HPeak (4,858)				72.1	77.7	98.2	82.0
FindPeaks (8,346)					77.7	91.6	87.1
ChIPseeqer (417)						58.5	75.8
SISSRs (2,455)							25.4
CisGenome (437)							

* We compare two sets of peaks (generated from two different peak-calling algorithms) to assess how much overlap can be found among them. Numbers displayed is the percentage of peaks in one set that are overlapped with at least one peak in another set. For each pair of peak sets, two percentages can be calculated by switching the order of the two sets. The higher percentage for each pair of peak sets is shown.

In addition, we observed that the patterns of the peaks identified from ChIP-Seq experiments on TFs like NRSF and STAT1 are different from those obtained from ChIP-Seq experiments on histone marks like H3K4me3 and H3K27me3. The average peak length is 253 bp for NRSF and 269 bp for STAT1 respectively. In contrast, peaks of H3K4me3 are much broader (738 bp) with high variance ((731 bp)^2^) while peaks of H3K27me3 are shorter (183 bp) with lower variance ((145 bp)^2^). Histograms of peak lengths from the four ChIP-Seq datasets can be found in Figure S3 in additional file 1.

## Discussion

In this study we have described HPeak, an HMM-based algorithm for defining ChIP-enriched peaks from short sequencing read data generated from ChIP-Seq experiments. Distinct from various algorithms currently available [10,11,20-31], HPeak explicitly assumes probability distributions to model coverage profiles of hypothetical DNA fragment (HDFs, see the Methods section) along the genome. After dividing each chromosome into bins, HPeak employs an HMM to distinguish ChIP-enriched regions from the background. Generalized Poisson (GP) [41] or zero inflated Poisson (ZIP) [42] distributions were used to model observed HDF counts in each bin, allowing for a more optimal fit to the data than a standard Poisson distribution. The end of each HDF was down-weighted when evaluating coverage to account for the uncertainties in ChIP DNA fragment length. These features facilitate the recognition of the core regions that show significant ChIP-enrichment. As a result, HPeak produces more calibrated peaks with higher motif concentration when compared to other peak-finding algorithms. Evaluation of experimental data showed favourable performance in terms of motif enrichment. Because the underlying HMM is quite general, HPeak may be applied to a wide spectrum of ChIP-Seq data with different experimental design and different sequencing depth, achieving balanced sensitivity and specificity with little or no fine-tuning by the users. In a recent study, Laajala et al. conducted a comprehensive performance comparison of existing peak-calling software [32]. HPeak was included in that study along with eight other published peak-calling algorithms. We noticed that overall HPeak performance is quite encouraging. For example, HPeak showed the best true positive rate and is closest to the optimum when testing on the NRSF ChIP-Seq data. This is consistent with what we have found.

A key advantage of model-based methods is that they are compatible with rigorous statistical inference. For example, under our model assumption, we can directly calculate the probability of observing the actual number of HDFs in a bin. Such probabilities can then be used to rank all peaks identified. This is important, as we have shown that higher-ranking peaks are more likely to harbour canonical binding motifs (Figure 1C). Furthermore, these probabilities can facilitate comparison of peaks across samples and studies. Another advantage of model-based method is that additional information such as GC content and mappability scores can be easily incorporated by extending the model.

In addition to its ability to identify the ChIP-enriched portion of the genome, HPeak provides more extensive information than other available programs. For example, as an option to users, HPeak provides more comprehensive annotation corresponding to each peak such as GC content, phylogenetic conservation (phastCons scores [43]), genomic features of the region (exon, intron, 5' UTR, 3' UTR, intergenic), and distance to the TSS of nearby genes. HPeak also provides an optional WIG file containing the genomic locations of all identified peaks, easily enabling the visualization of all of the peaks within the UCSC genome browser. Further, HPeak provides an optional FASTA format sequence file containing nucleotide sequences of all peaks to facilitate subsequent motif analysis.

When comparing publicly available STAT1 ChIP-Seq and ChIP-chip data, we found that the ChIP-Seq technique has a clear advantage over the ChIP-chip technique in terms of enriching for an expected motif under the predicted peaks. The improvement can be largely attributed to the increased resolution offered by the new sequencing technology. By enabling the detection of narrower peaks flanking the true binding sites, ChIP-Seq reveals a higher concentration of the predicted binding motif within its peaks. It is known that the significance measure derived from the ChIP-chip data is correlated with the probability that a region contains the canonical binding motif [40]. We found that such correlation is much stronger in ChIP-Seq data (Figure 1C). This implies that the read coverage profile is very informative on the presence and location of actual functional binding sites.

In this study, rather than the commonly used Chi-square goodness-of-fit test, we proposed to use the K-S test as an alternative to evaluate the reproducibility of datasets obtained under different conditions. We found that the K-S test is better suited for sequencing data than the Chi-square goodness-of-fit test, since there is no need to divide chromosomes into windows and correlation/reproducibility can be conveniently visualized by plotting ECDFs (Figure S1 in additional file 1).

It is worth pointing out that it is challenging to determine criteria to evaluate the performance of various peak-calling algorithms on experimental ChIP-Seq data. This is because in general very little information is available on true positive and true negative loci. We choose to use the prevalence of known motifs as a metric for performance. One caveat of this approach is that, as pointed out in Hu et al. 2010 [44], many of the motif patterns stored in the database may not be accurate and there maybe novel motifs that do not exist in motif databases. We speculate that the inaccurate motif patterns will affect the results of all peak-calling algorithms equally, but the actual effect remains to be seen and further studies seem warranted. Additionally, our method is not well-suited for quantifying false detection rate therefore some methods maybe put in a disadvantaged position in our comparison. Because of this, our performance evaluation results should be interpreted with caution.

The ChIP-Seq technology can be applied to other types of proteins in addition to TFs. For example, multiple studies have utilized ChIP-Seq to identify histone modification sites in the genome [12-14], which is reviewed in Park [45]. Since the underlying two-component HMM is quite general, HPeak can also be applied to data collected from other types of sequencing-based experiments such as MeDIP-seq [46], RNA-seq [47] and methylation pattern discovery [19]. In these experiments, HPeak can be used to identify regions in the genome that is significantly enriched for sequencing reads. Some adjustment of HPeak parameters such as bin size may be needed when analyzing non-ChIP-Seq type of data.

The HMM used in HPeak assumes two different states: enriched and non-enriched. Although such a scheme is well-accepted in ChIP-chip analyses [33], it is possible and of interest to consider more sophisticated HMM schemes where more than two states are allowed. As an example, we may consider a four-state HMM: enrichment of reads on the positive strand, enrichment of reads on the negative strand, enrichment of reads on both strands and no enrichment. By utilizing strand information, we will be able to better identify true binding events since a symmetric pattern among reads with different strands is expected around the binding sites. Another possibility is to distinguish shapes of peaks, such as sharp peaks, broad and low plateaus. These may help us to distinguish different types of binding events.

We assume HDF counts follow ZIP and GP distributions in background and ChIP-enriched regions respectively. Other probability distributions such as negative binomial (NB) has also been used to model ChIP-Seq data [22,28]. It is of interest to understand whether these distributions fit observed ChIP-Seq data better than the standard Poisson distribution. To investigate, we fit GP, Poisson and NB distributions to the number of HDFs found in NRSF and STAT1 ChIP-Seq data. For the number of HDFs found in the ChIP-enriched regions, we found that the GP distribution shows a much better fit than the Poisson distribution and a slightly better fit than the NB distributions. An example of the model fit can be found in Figure S4 in additional file 1. For the number of HDFs found in background regions, we found that the ZIP distribution produces a slightly better fit than both Poisson and NB distributions (data not shown).

The current HPeak algorithm does not distinguish reads of different orientation within a peak. Such information has been shown to be informative in pinpointing the summit of the peak and to estimate the DNA fragment length [20,21,25,48]. We plan to incorporate such information in the future release of HPeak and we believe it will further enhance the performance of the HPeak program.

## Conclusions

Based on our study, we believe that HPeak will be highly useful to researchers conducting ChIP-Seq experiments. For instance, HPeak has already been utilized in a recent study to map the genomic landscape of master transcriptional regulators of prostate cancer [49]. We envision that this tool will greatly facilitate the rapid and accurate analysis of the emerging ChIP-Seq data generated by the research community.

## Methods

### HPeak scheme

**Figure 3:**
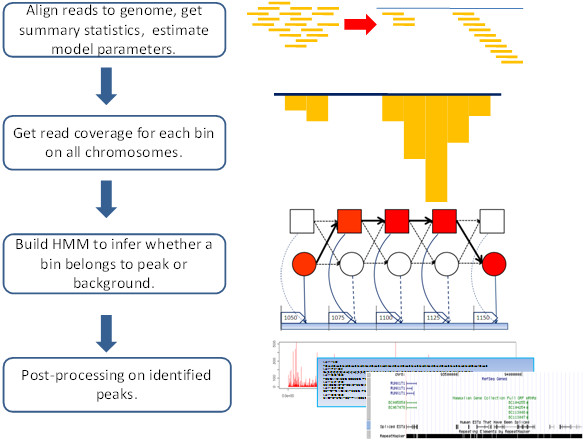
**Workflow of HPeak analysis of ChIP-Seq data**.

The goal of ChIP-Seq analysis is to partition the genome into ChIP-enriched and non-enriched segments based on the number of mapped sequencing reads, such that the enriched portion of the genome is much more likely to harbour protein-DNA interaction sites. The entire procedure of HPeak analysis can be summarized into four steps (Figure 3).

In the initial step, HPeak imports the genomic coordinates of all mapped sequencing reads. Various ELAND output format (Illumina Inc, San Diego, CA) and BED formats are allowed. In order to represent the entire DNA fragment, we followed the method used by Robertson et al. [11] to extend each short read directionally from its start position to form a hypothetical DNA fragment (HDF), mimicking the ChIP DNA fragment from which the sequencing read was generated.

In the second step, HPeak partitions the entire genome into small bins of fixed length (25 bp in this study) and subsequently counts the numbers of HDFs that fall in each bin throughout the genome. By adopting a bin approach, we are able to simplify computation and facilitate straightforward comparison across samples while obtaining a high resolution genome-wide ChIP DNA coverage profile. The size of the bins can be adjusted to balance the computational run time with resolution.

In the third step, HPeak applies a two-state HMM on the HDF coverage profile to distinguish blocks of consecutive ChIP-enriched bins from the background. GP and ZIP distributions were utilized to model the numbers of HDFs found in ChIP-enriched and non-enriched bins.

In the last step, HPeak generates additional output files based on the peaks called in the previous step. These include a wiggle (WIG) format file for visualization, a sequence file for subsequent motif analysis and an annotation file that details the genomic properties of each peak identified.

### Down-weighting the end of HDF to account for length uncertainty

In ChIP-Seq experiments, a size selection step is employed during sample preparation to restrict the size of DNA fragments to be sequenced to a certain range (for example, 200 bp ± 25 bp). Since only the beginning or end portion of each DNA fragment is sequenced, we employ a read extension step described earlier in order to quantify the actual DNA fragment coverage at any given locus. To account for the uncertainties in the lengths of the DNA fragments, we gradually down-weight the HDF coverage contribution in bins covered by the end portion of each HDF. For example, if the DNA fragments are size-selected to be between 175 and 225 bp, we assign one unit of coverage to the genomic locations covered by the first 175 bp of the HDF, linearly decreasing the coverage assigned to the last 50 bp from 1 to 0 (Figure S5A in additional file 1). We also assign partial coverage to bins with partial HDF coverage.

### Statistical model to define ChIP-enriched regions

We adopted a two-state HMM to model the observed ChIP DNA fragment profile and classify the bins into either ChIP-enriched (peaks) or non-enriched (background) along the entire genome (Figure S5B in additional file 1). We employ a HMM because of the strong correlation of HDF coverage in adjacent bins. To infer the emission probabilities in the HMM, we used GP and ZIP distributions to model the numbers of HDFs found in the two types of bins. Both distributions were modified from and are more flexible than the standard Poisson distribution. The GP distribution was adopted for the ChIP-enriched group. Due to the wide dynamic range of ChIP-Seq data, the number of HDFs falling into ChIP-enriched bins varied dramatically and showed significant over-dispersion (the variances of HDF counts in ChIP-enriched bins are about ten times larger than the mean HDF counts in these bins). A standard Poisson distribution requires the mean and variance to be the same which is unrealistic for this type of data. In contrast, the GP distribution is much more flexible, since it contains two parameters and allows the variance to be greater than the mean. The probability density function of the GP distribution is

here *ϕ*, *λ *> 0.

Since the majority of the genome is not enriched, there are significantly more empty bins required to model the background than would be expected from the Poisson distribution. An ideal alternative is the zero-inflated Poisson (ZIP) distribution [42], which is essentially a mixture distribution of point mass at zero and a Poisson distribution. The probability density function of the ZIP is:

where *π *(0 ≤ *π *≤ 1) is the proportion of zeros in the mixture distribution. Compared to the standard Poisson distribution, the GP and ZIP distributions provide a much better fit to the observed data due to improved flexibility. This allows the assignment of accurate probabilities to each bin and defines the boundaries of the enriched regions more precisely.

Some ChIP-Seq experiments are carried out using paired samples: a treated sample (stimulated) with an untreated control (unstimulated). For the paired design, we used the bivariate GP/ZIP distributions to model the difference in HDF coverage in the two types of samples. More specifically, let *D*_*i *_= *X*_*i *_- *Y*_*i*_, *i *= 1,2. *X*_*i*_, *Y*_*i *_represent the HDF counts in bins residing in the treated and untreated samples respectively; *i *= 1 indicates ChIP-enriched; *i *= 2 indicates nonenriched. Assuming that *X*_1 _follows the GP distribution while *X*_2 _*Y*_1 _and *Y*_2 _follow ZIP distributions, we calculate the marginal probabilities of observing the HDF coverage differences between the two samples in the same bin, based on the bivariate distributions using the parameters estimated within treated or untreated samples separately. An HMM is then designed to perform inference on the enriched/non-enriched states.

### HMM parameter estimation

For HMM parameter estimation, we use the well-established Viterbi algorithm [50]. In the initial step, we use summary statistics to get a quick and rough estimate of the transition and emission probabilities. Following the method described in Robertson et al. [11], all HDFs that overlap are merged into a single consecutive candidate peak. The read coverage of each candidate peak is then calculated to determine whether it surpasses a significance threshold that is required for it to be classified as a ChIP-enriched peak. The initial probability of being in a peak, and the transition probability from background to peaks, is equivalent to the proportion of the genome that is covered by the peaks, as defined above. The transition probability from a peak back to the background is defined such that the *a priori *length of the peaks is roughly equivalent to the median length of the peaks as defined above. For instance, supposing that *L *represents the median length of these peaks, *p*_*0 *_represents the transition probability from peak to background, and *d *is the bin size, then *p*_*0 *_can be estimated by solving (1 - *p*_0_)^*L*/*d *^= 1/2.

The emission probabilities in the HMM are derived from the GP and ZIP distributions that are used to model the HDF count data in ChIP-enriched and non-enriched bins. The initial parameters in these distributions were estimated as follows. For the ZIP distribution that models background HDF counts, we first selected the bins with HDF counts less than *k*_*low*_, where *k*_*low *_= *k*/2 and *k *is the minimum number of HDFs found in bins belonging to ChIP-enriched peaks. Next, we used the method of moments to estimate GP distribution parameters *ϕ *and *λ*, as well as ZIP distribution parameters *μ *and *π*. Detailed formulas of these estimation can be found in additional file 1. For the two-sample case, we obtain the parameters of two sets of GP and ZIP distributions from treated and untreated samples separately.

After the initial step, we iterate the two steps of the Viterbi algorithm: conditional on the current estimate of the model parameters, we derive the hidden states for each bin across the genome by evaluating which model fits the observed HDF count data better; conditional on the currently assigned hidden states, we separate all bins into ChIP-enriched and non-enriched, and update model parameters for the two categories separately. Here we no longer merge HDFs; all hidden state inferences are conducted at the bin level.

As a final result, using a user-specified posterior probability threshold, we identify blocks of consecutive bins that show significant enrichment of HDF counts from the HMM. Each set of bins is then defined as a peak. In addition to its genomic location and the length of the peak, HPeak reports the location of the highest HDF coverage within the peak, the actual maximum HDF coverage at that location, and the log transformed posterior probability of these bins being ChIP-enriched. These probabilities reflect the significance of these peaks and therefore can be used to rank the peaks.

### Implementation

We have implemented HPeak in a software package using Perl and C++. HPeak can run on most platforms including Linux, Windows and Mac OS. Using a Dell PowerEdge computer server, we found that HPeak required reasonable amounts of memory (less than 2G for a dataset of 15 million reads) and time (approximately 30 and 60 minutes for treated only and paired design respectively). Currently, HPeak is capable of analyzing ChIP-Seq data collected from human and mouse and can easily be extended to other species. The HPeak program is freely available at http://www.sph.umich.edu/csg/qin/HPeak.

### Existing peak detection algorithm

We provide a short summary for each of the algorithms being compared in this study. For more details, please consult the original publications or the software websites.

#### Peakfinder (ChIPSeq-mini)

This is perhaps the earliest software for ChIP-Seq peak calling. A candidate peak is called if it contains at least *k *reads not separated by more than *n *bp (75 by default), and at least five of these reads are overlapping. An additional requirement is that there is at least *m*-fold enrichment (default 5-fold) when control samples are available.

#### FindPeaks

Findpeaks extends each read directionally to form an HDF. The length of the extension is taken to be the average length of the size-selected ChIP-DNA fragments. Subsequently overlapping HDFs are merged to form a candidate peak. A binding site is then identified if the number of HDFs in a candidate peak exceeds the significance threshold. An FDR for each binding site is estimated based on Monte Carlo simulation, which is the number of peaks identified in the randomized data divided by the number of peaks identified in the real data under the same significance threshold.

#### MACS

MACS first separate reads of different strands, and then empirically models the shift between the two types of strands. MACS also implements a Poisson distribution-based model to characterize the background read distribution. They termed their model a "dynamic Poisson model" to reflect the fact that the parameter of the Poisson distribution is allowed to fluctuate along the genome in order to capture local sequencing bias. MACS works with or without negative control samples. When negative control samples are available, an FDR is estimated by dividing the number of peaks identified in the control sample by the number of peaks identified in the ChIP sample.

#### CisGenome

CisGenome employs a sliding window strategy to identify regions with over-abundant reads. The authors assume a NB distribution for the background read occurrence which was said to provide a better fit of the data than the Poisson distribution. When negative control data are available, the authors use a binomial model to decide whether the enrichment of reads in the ChIP channel is significantly higher than in the control.

#### SISSRs

SISSRs first extends each read to form an HDF, next partitions the genome into windows of equal sizes, then scans the genome to count the number of reads landing in each window. Binding sites are called when the majority of reads switch from one strand to the other. A negative control sample, or a Poisson background model is used to estimate the FDR, defined to be the ratio of the number of peaks in the control sample or background model to the number of peaks observed.

#### ChIPseeqer (revised from the description provided by Dr. Elemento)

In this program, a read density map is first constructed by extending the reads to the average length of the DNA fragments in the sequenced DNA library and by counting the number of overlapping reads at each nucleotide position. The Poisson distribution probability model is then used to compare the observed read count to the expected read count for both ChIP and input (control) data (if available) and to compute a normalized peak score for each nucleotide position (based on probabilities). A peak is called when this score is greater than a specific threshold (default 15) and the interval is at least 100 bp. It is important to note that the algorithm uses the mappability map to correct for the variation in mappablity between sequences. Moreover, if input data are provided, it is further required that there is at least an m-fold enrichment (default 2-fold) of reads in the ChIP data over the input.

#### ChIPDiff

ChIPDiff was developed to identify differential histone modification sites genome-wide. This method employs an HMM to infer the states of histone modification changes at each genomic location based on the observed ChIP fragment counts. To apply ChIPDiff, it is required that ChIP-Seq data from two sources (two different cell lines, etc) are available.

### Performance evaluation metrics

#### Motif enrichment analysis

Since consensus motifs are often enriched in the binding sites of the TF, motif enrichment may serve as a measure of the performance (sensitivity and specificity) when comparing ChIP-Seq peak-calling algorithms. One strategy is to scan all sequences identified as ChIP-enriched (referred to as test sequences) and compare the proportion of sequences that contain the motif of interest. However, longer sequences, by chance, are more likely to contain motifs of interest. To adjust for this bias, we introduced length-matched control sequences. These random control sequences were extracted from a collection of the regions 5 kb upstream of annotated transcription starts of all RefSeq genes with annotated 5 UTRs. This set of promoter sequences was downloaded from the UCSC genome browser [51] website http://hgdownload.cse.ucsc.edu/downloads.html. Any sequence that overlapped with test sequences was excluded. Motif scan of the test and the control sequences was then performed using MatInspector [52] in the Genomatix suite (Genomatix GmBH, Munich, Germany) with default settings. The numbers of test and control sequences either harbouring or lacking the expected motifs were recorded in a 2 × 2 contingency table. A Chi-square test was then performed to evaluate the significance of the motif enrichment. Motif enrichments of all 153 families of vertebrate motif matrices found in the Genomatix MatBase 7.0 database were calculated then ranked by the Chi-square test statistics.

## Authors' contributions

ZSQ, JY, JS developed the methods and coded the software package. MH participated in the analysis of real datasets. CAM, SK, JY and AMC participated in the design and coordination of the study and reviewed the manuscript. JY and AMC also provided key biological insight that benefits the study design. All authors have read and approved the final manuscript.

## Supplementary Material

Additional file 1 **More detailed information on methods and additional Tables and Figures**. Click here for file

## References

[B1] Orlando V Paro R Mapping Polycomb-repressed domains in the bithorax complex using in vivo formaldehyde cross-linked chromatin Cell 1993 75 1187 1198 10.1016/0092-8674(93)90328-N 7903220

[B2] Solomon MJ Larsen PL Varshavsky A Mapping protein-DNA interactions in vivo with formaldehyde: evidence that histone H4 is retained on a highly transcribed gene Cell 1988 53 937 947 10.1016/S0092-8674(88)90469-2 2454748

[B3] Massie CE Mills IG ChIPping away at gene regulation EMBO Rep 2008 9 337 343 10.1038/embor.2008.44 18379585 PMC2288763

[B4] Ren B Robert F Wyrick JJ Aparicio O Jennings EG Simon I Zeitlinger J Schreiber J Hannett N Kanin E Genome-wide location and function of DNA binding proteins Science 2000 290 2306 2309 10.1126/science.290.5500.2306 11125145

[B5] Lieb JD Liu X Botstein D Brown PO Promoter-specific binding of Rap1 revealed by genome-wide maps of protein-DNA association Nat Genet 2001 28 327 334 10.1038/ng569 11455386

[B6] Kim TH Barrera LO Ren B ChIP-chip for genome-wide analysis of protein binding in mammalian cells Curr Protoc Mol Biol 2007 Chapter 21 Unit 21 13 18265397 10.1002/0471142727.mb2113s79

[B7] Mardis ER ChIP-seq: welcome to the new frontier Nat Methods 2007 4 613 614 10.1038/nmeth0807-613 17664943

[B8] Barski A Zhao K Genomic location analysis by ChIP-Seq J Cell Biochem 2009 107 11 18 10.1002/jcb.22077 19173299 PMC3839059

[B9] Schmidt D Wilson MD Spyrou C Brown GD Hadfield J Odom DT ChIP-seq: Using high-throughput sequencing to discover protein-DNA interactions Methods 2009 19275939 10.1016/j.ymeth.2009.03.001 PMC4052679

[B10] Johnson DS Mortazavi A Myers RM Wold B Genome-wide mapping of in vivo protein-DNA interactions Science 2007 316 1497 1502 10.1126/science.1141319 17540862

[B11] Robertson G Hirst M Bainbridge M Bilenky M Zhao Y Zeng T Euskirchen G Bernier B Varhol R Delaney A Genome-wide profiles of STAT1 DNA association using chromatin immunoprecipitation and massively parallel sequencing Nat Methods 2007 4 651 657 10.1038/nmeth1068 17558387

[B12] Mikkelsen TS Ku M Jaffe DB Issac B Lieberman E Giannoukos G Alvarez P Brockman W Kim TK Koche RP Genome-wide maps of chromatin state in pluripotent and lineage-committed cells Nature 2007 448 553 560 10.1038/nature06008 17603471 PMC2921165

[B13] Barski A Cuddapah S Cui K Roh TY Schones DE Wang Z Wei G Chepelev I Zhao K High-resolution profiling of histone methylations in the human genome Cell 2007 129 823 837 10.1016/j.cell.2007.05.009 17512414

[B14] Schones DE Cui K Cuddapah S Roh TY Barski A Wang Z Wei G Zhao K Dynamic regulation of nucleosome positioning in the human genome Cell 2008 132 887 898 10.1016/j.cell.2008.02.022 18329373 PMC10894452

[B15] Chen X Xu H Yuan P Fang F Huss M Vega VB Wong E Orlov YL Zhang W Jiang J Integration of external signaling pathways with the core transcriptional network in embryonic stem cells Cell 2008 133 1106 1117 10.1016/j.cell.2008.04.043 18555785

[B16] Lefrancois P Euskirchen GM Auerbach RK Rozowsky J Gibson T Yellman CM Gerstein M Snyder M Efficient yeast ChIP-Seq using multiplex short-read DNA sequencing BMC Genomics 2009 10 37 10.1186/1471-2164-10-37 19159457 PMC2656530

[B17] Welboren WJ van Driel MA Janssen-Megens EM van Heeringen SJ Sweep FC Span PN Stunnenberg HG ChIP-Seq of ERalpha and RNA polymerase II defines genes differentially responding to ligands Embo J 2009 28 1418 1428 10.1038/emboj.2009.88 19339991 PMC2688537

[B18] Visel A Blow MJ Li Z Zhang T Akiyama JA Holt A Plajzer-Frick I Shoukry M Wright C Chen F ChIP-seq accurately predicts tissue-specific activity of enhancers Nature 2009 457 854 858 10.1038/nature07730 19212405 PMC2745234

[B19] Brunner AL Johnson DS Kim SW Valouev A Reddy TE Neff NF Anton E Medina C Nguyen L Chiao E Distinct DNA methylation patterns characterize differentiated human embryonic stem cells and developing human fetal liver Genome Res 2009 19 1044 1056 10.1101/gr.088773.108 19273619 PMC2694474

[B20] Zhang Y Liu T Meyer CA Eeckhoute J Johnson DS Bernstein BE Nussbaum C Myers RM Brown M Li W Liu XS Model-based Analysis of ChIP-Seq (MACS) Genome Biol 2008 9 R137 10.1186/gb-2008-9-9-r137 18798982 PMC2592715

[B21] Valouev A Johnson DS Sundquist A Medina C Anton E Batzoglou S Myers RM Sidow A Genome-wide analysis of transcription factor binding sites based on ChIP-Seq data Nat Methods 2008 5 829 834 10.1038/nmeth.1246 19160518 PMC2917543

[B22] Ji H Jiang H Ma W Johnson DS Myers RM Wong WH An integrated software system for analyzing ChIP-chip and ChIP-seq data Nat Biotechnol 2008 26 1293 1300 10.1038/nbt.1505 18978777 PMC2596672

[B23] Jothi R Cuddapah S Barski A Cui K Zhao K Genome-wide identification of in vivo protein-DNA binding sites from ChIP-Seq data Nucleic Acids Res 2008 36o 5221 5231 10.1093/nar/gkn488 PMC2532738 18684996

[B24] Xu H Wei CL Lin F Sung WK An HMM approach to genome-wide identification of differential histone modification sites from ChIP-seq data Bioinformatics 2008 24 2344 2349 10.1093/bioinformatics/btn402 18667444

[B25] Kharchenko PV Tolstorukov MY Park PJ Design and analysis of ChIP-seq experiments for DNA-binding proteins Nat Biotechnol 2008 26 1351 1359 10.1038/nbt.1508 19029915 PMC2597701

[B26] Rozowsky J Euskirchen G Auerbach RK Zhang ZD Gibson T Bjornson R Carriero N Snyder M Gerstein MB PeakSeq enables systematic scoring of ChIP-seq experiments relative to controls Nat Biotechnol 2009 27 66 75 10.1038/nbt.1518 19122651 PMC2924752

[B27] Nix DA Courdy SJ Boucher KM Empirical methods for controlling false positives and estimating confidence in ChIP-Seq peaks BMC Bioinformatics 2008 9 523 10.1186/1471-2105-9-523 19061503 PMC2628906

[B28] Spyrou C Stark R Lynch AG Tavare S BayesPeak: Bayesian analysis of ChIP-seq data BMC Bioinformatics 2009 10 299 10.1186/1471-2105-10-299 19772557 PMC2760534

[B29] Choi H Nesvizhskii AI Ghosh D Qin ZS Hierarchical hidden Markov model with application to joint analysis of ChIP-chip and ChIP-seq data Bioinformatics 2009 25 1715 1721 10.1093/bioinformatics/btp312 19447789 PMC2732365

[B30] Fejes AP Robertson G Bilenky M Varhol R Bainbridge M Jones SJ FindPeaks 3.1: a tool for identifying areas of enrichment from massively parallel short-read sequencing technology Bioinformatics 2008 24 1729 1730 10.1093/bioinformatics/btn305 18599518 PMC2638869

[B31] Albert I Wachi S Jiang C Pugh BF GeneTrack--a genomic data processing and visualization framework Bioinformatics 2008 24 1305 1306 10.1093/bioinformatics/btn119 18388141 PMC7058423

[B32] Laajala TD Raghav S Tuomela S Lahesmaa R Aittokallio T Elo LL A practical comparison of methods for detecting transcription factor binding sites in ChIP-seq experiments BMC Genomics 2009 10 618 10.1186/1471-2164-10-618 20017957 PMC2804666

[B33] Li W Meyer CA Liu XS A hidden Markov model for analyzing ChIP-chip experiments on genome tiling arrays and its application to p53 binding sequences Bioinformatics 2005 21 Suppl 1 i274 282 10.1093/bioinformatics/bti1046 15961467

[B34] Ji H Wong WH TileMap: create chromosomal map of tiling array hybridizations Bioinformatics 2005 21 3629 3636 10.1093/bioinformatics/bti593 16046496

[B35] Munch K Gardner PP Arctander P Krogh A A hidden Markov model approach for determining expression from genomic tiling micro arrays BMC Bioinformatics 2006 7 239 10.1186/1471-2105-7-239 16672042 PMC1481622

[B36] Huber W Toedling J Steinmetz LM Transcript mapping with high-density oligonucleotide tiling arrays Bioinformatics 2006 22 1963 1970 10.1093/bioinformatics/btl289 16787969

[B37] Humburg P Bulger D Stone G Parameter estimation for robust HMM analysis of ChIP-chip data BMC Bioinformatics 2008 9 343 10.1186/1471-2105-9-343 18706106 PMC2536674

[B38] Euskirchen GM Rozowsky JS Wei CL Lee WH Zhang ZD Hartman S Emanuelsson O Stolc V Weissman S Gerstein MB Mapping of transcription factor binding regions in mammalian cells by ChIP: comparison of array- and sequencing-based technologies Genome Res 2007 17 898 909 10.1101/gr.5583007 17568005 PMC1891348

[B39] Benjamini Y Hochberg Y Controlling the false discovery rate: a pratical and powerful approach to multiple testing J Royal Stat Soc B 1995 57 289 300

[B40] Shim H Keles S Integrating quantitative information from ChIP-chip experiments into motif finding Biostatistics 2008 9 51 65 10.1093/biostatistics/kxm014 17533175

[B41] Consul PC Generalized Poisson Distributions 1989 New York: Marcel Dekker

[B42] Johnson NL Kotz S Kemp AW Univariate discrete distributions 1992 2 New York: John Wiley & Sons

[B43] Siepel A Bejerano G Pedersen JS Hinrichs AS Hou M Rosenbloom K Clawson H Spieth J Hillier LW Richards S Evolutionarily conserved elements in vertebrate, insect, worm, and yeast genomes Genome Res 2005 15 1034 1050 10.1101/gr.3715005 16024819 PMC1182216

[B44] Hu M Yu J Taylor JM Chinnaiyan AM Qin ZS On the detection and refinement of transcription factor binding sites using ChIP-Seq data Nucleic Acids Res 2010 38 2154 2167 10.1093/nar/gkp1180 20056654 PMC2853110

[B45] Park PJ Epigenetics meets next-generation sequencing Epigenetics 2008 3 10.4161/epi.3.6.7249 19098449

[B46] Down TA Rakyan VK Turner DJ Flicek P Li H Kulesha E Graf S Johnson N Herrero J Tomazou EM A Bayesian deconvolution strategy for immunoprecipitation-based DNA methylome analysis Nat Biotechnol 2008 26 779 785 10.1038/nbt1414 18612301 PMC2644410

[B47] Mortazavi A Williams BA McCue K Schaeffer L Wold B Mapping and quantifying mammalian transcriptomes by RNA-Seq Nat Methods 2008 5 621 628 10.1038/nmeth.1226 18516045 PMC13303166

[B48] Valouev A Johnson DS Sundquist A Medina C Anton E Batzoglou S Myers RM Sidow A Genome-wide analysis of transcription factor binding sites based on ChIP-Seq data Nat Methods 2008 19160518 10.1038/nmeth.1246 PMC2917543

[B49] Yu J Yu J Mani RS Cao Q Brenner CJ Cao X Wang X Wu L Li J Hu M An integrated network of androgen receptor, polycomb, and TMPRSS2-ERG gene fusions in prostate cancer progression Cancer Cell 17 443 454 10.1016/j.ccr.2010.03.018 20478527 PMC2874722

[B50] Rabiner LR A Tutorial On Hidden Markov-Models and Selected Applications in Speech Recognition Proceedings of the Ieee 1989 77 257 286 10.1109/5.18626

[B51] Kent WJ Sugnet CW Furey TS Roskin KM Pringle TH Zahler AM Haussler D The human genome browser at UCSC Genome Res 2002 12 996 1006 12045153 10.1101/gr.229102 PMC186604

[B52] Cartharius K Frech K Grote K Klocke B Haltmeier M Klingenhoff A Frisch M Bayerlein M Werner T MatInspector and beyond: promoter analysis based on transcription factor binding sites Bioinformatics 2005 21 2933 2942 10.1093/bioinformatics/bti473 15860560

