# Supplementary material for: HPeak: an HMM-based algorithm for defining read-enriched regions in ChIP-Seq data
Source: BMC Bioinformatics. 2010 Jul 2;11:369. doi: 10.1186/1471-2105-11-369 (PMC2912305; doi:10.1186/1471-2105-11-369)

HPeak: An HMM-based algorithm for defining read-enriched regions in ChIP-Seq data

Zhaohui Qin, Jianjun Yu, Jincheng Shen, Christopher A. Maher, Ming Hu, Shanker Kalyana-Sundaram, Jindan Yu, Arul M. Chinnaiyan

Supplementary Material

Initial estimate of parameters in the GP and ZIP distributions

For the GP distribution to model HDF counts inside a peak, its parameters were estimated based on number of bins containing at least *k* HDFs. Since

$$E(X) = \lambda$$

and

$$Var(X) = \lambda(1 + \phi\lambda)^2.$$

Method of moment gives estimator of α and λ as:

$$\hat{\lambda} = \bar{X} \text{ and } \hat{\phi} = \left(\sqrt{\frac{S^2}{\hat{\lambda}}} - 1\right)/\hat{\lambda}.$$

For the ZIP distribution, since $E(X) = \bar{X}$, $S^2 = E(X)(1 + \mu - E(X))$, we have

$$\hat{\mu} = \frac{S^2}{\bar{X}} - 1 + \bar{X} \text{ and } \hat{\pi} = \frac{\bar{X}}{\hat{\mu}}.$$

Table S1. Summary of overlaps among peaks identified by different peaking calling algorithms in NRSF and STAT1 ChIP-seq datasets*.

NRSF

	Peak Finder	MACS	HPeak	Find Peaks	HPeak (chip)	ChIP seeqer	SISSRs	Cis Genome
Peak Finder 1,935		100	100	100	100	98.3	99.9	98.7
MACS 4,679			83.3	87.4	90.2	99.7	87.4	97.8
HPeak 4,404				91.7	88.7	100	87.3	98.6
FindPeaks 3,445					98.0	100	89.3	99.2
HPeak (chip) 4,085						100	86.3	99.2
ChIPseeqer 2,361							98.9	94.8
SISSRs 5,243								95.8
CisGenome 2,545								

STAT1

	Peak Finder	MACS	HPeak	Find Peaks	HPeak (chip)	ChIP seeqer	SISSRs	Cis Genome
Peak Finder								
MACS 22,402			78.4	85.1	87.8	92.7	94.7	78.1
HPeak 24,490				95.4	98.4	99.9	98.5	85.4
FindPeaks 41,127					94.1	100	97.5	92.5
HPeak (chip) 43,443						100	99.7	92.7
ChIPseeqer 11,662							66.0	100
SISSRs 9,561								87.3
CisGenome 38,878								

* We compare two sets of peaks (generated from two different peak-calling algorithms) to assess how much overlap can be found among them. Numbers displayed is the percentage of peaks in one set that are overlapped with at least one peak in another set. For each pair of peak sets, two percentages can be calculated by switching the order of the two sets. The higher percentage for each pair of peak sets is shown.

Figure S1. Evaluation of the reproducibility of the ChIP-Seq assay. We plot pairs of empirical cumulative density functions (ECDFs, step function) of read start locations on each chromosome. For each plot, similarity of the curves indicates resemblance of the two reads distribution, hence reproducibility. **A.** ECDFs of reads from the STAT1 stimulated sample loaded on lanes 1 (blue) and 2 (red). **B.** ECDFs of reads from STAT1 stimulated sample land 1 (blue) and unstimulated sample lane 3 (red).

A.

Positive strand:

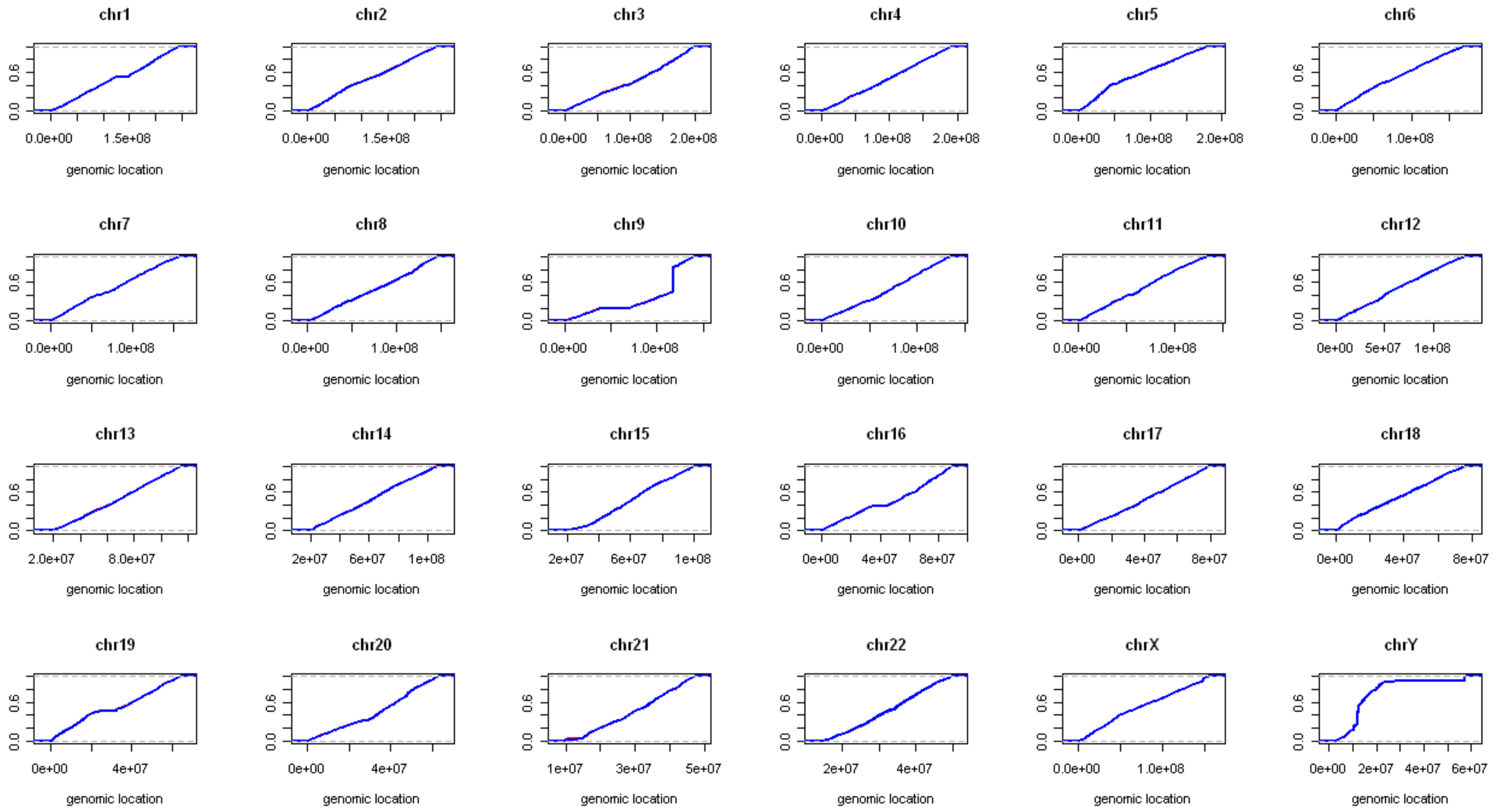

Negative strand:

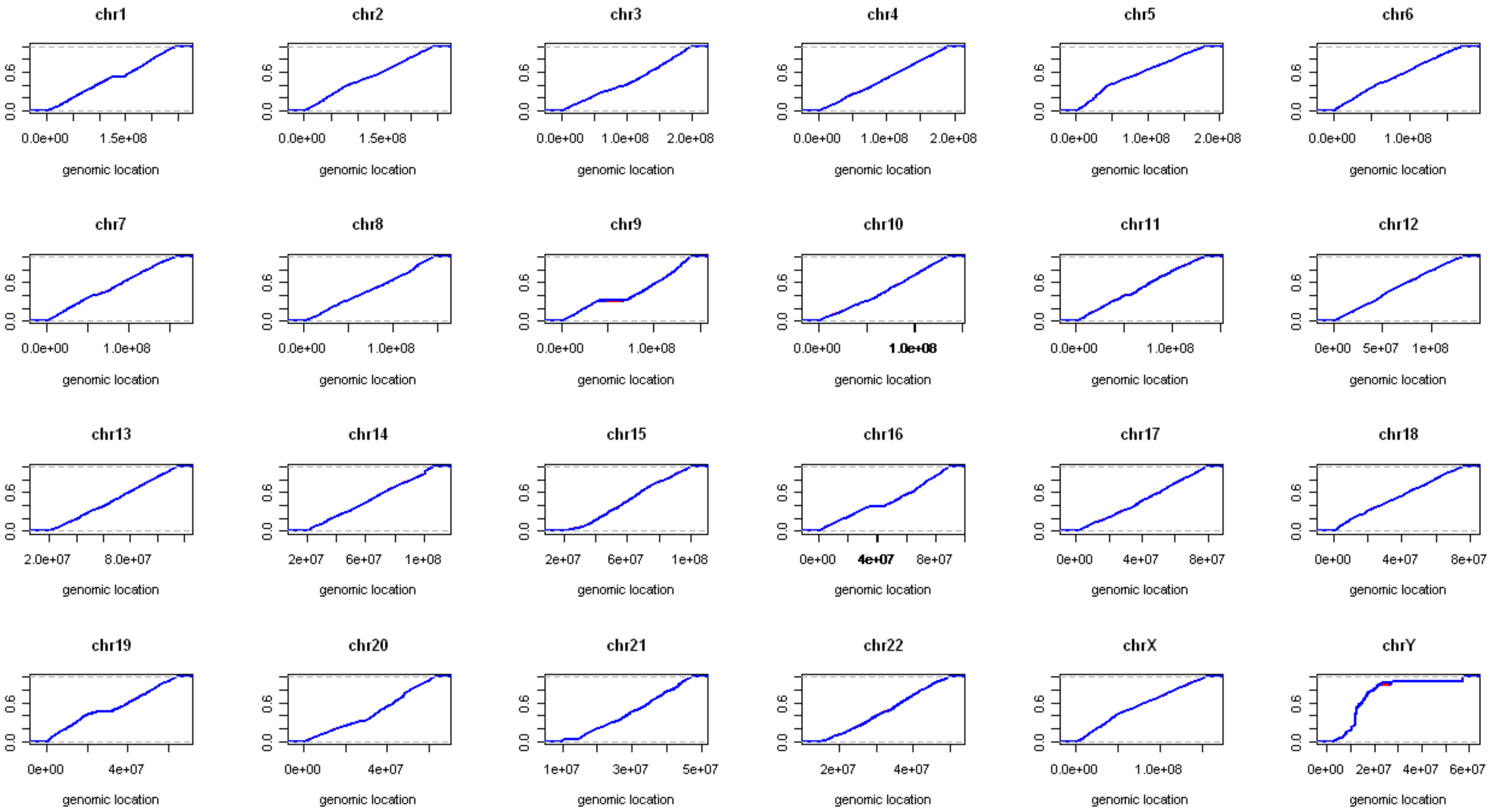

B.
Positive strand:

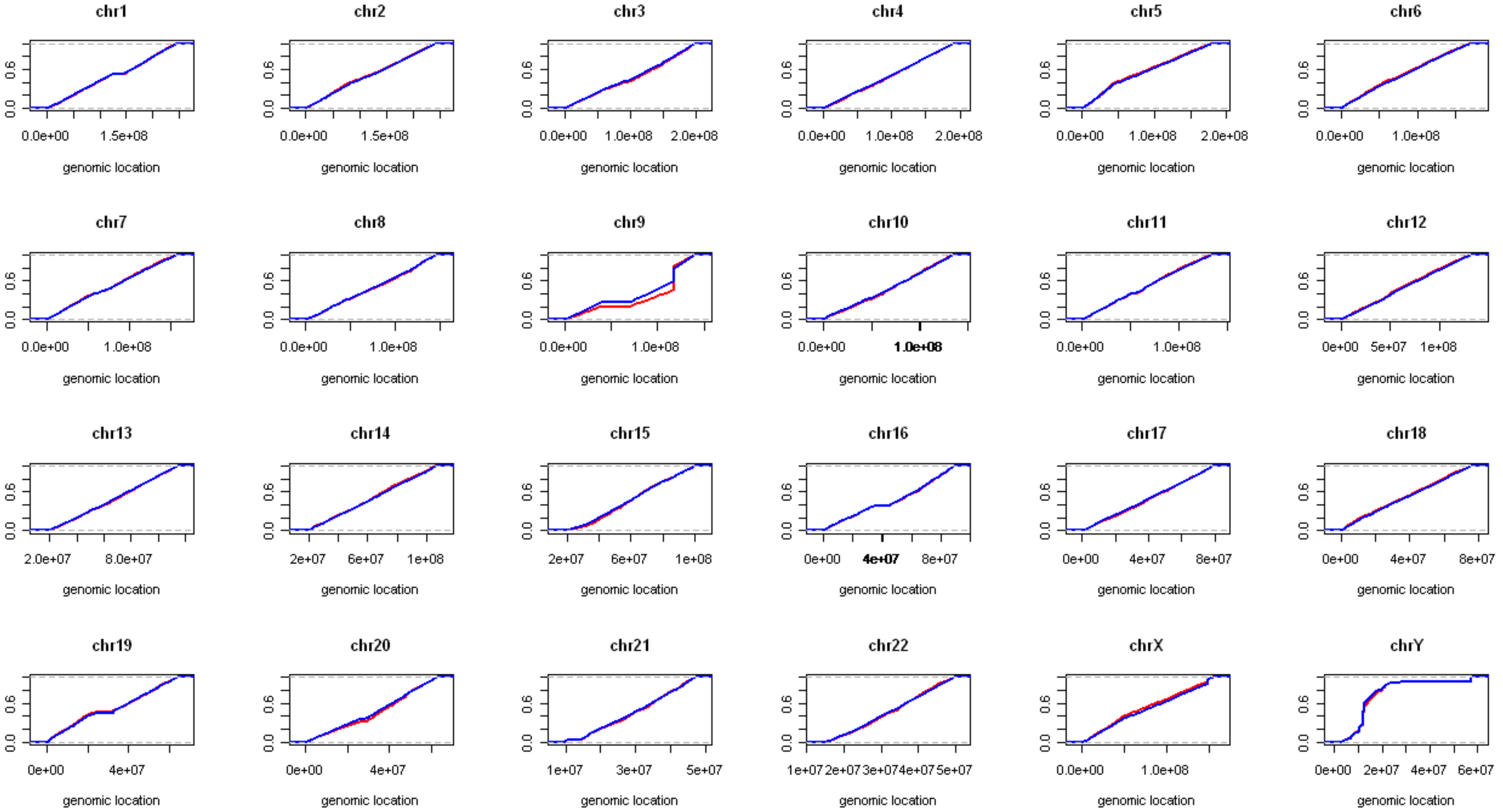

Negative strand:

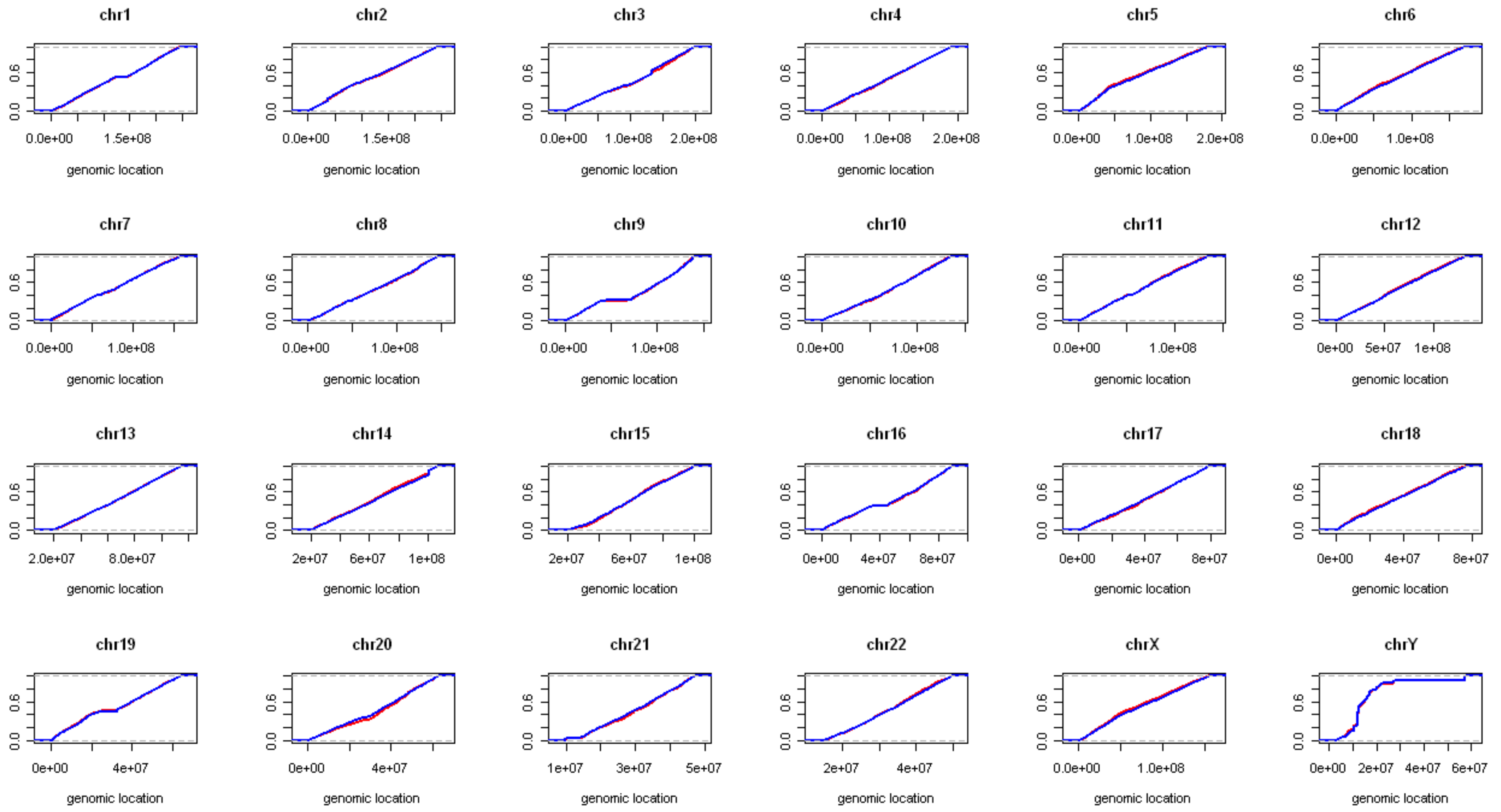

Figure S2. Performance comparison between HPeak (using data from both treated and untreated samples or using data from treated sample only) and other ChIP-Seq analysis algorithms. We use the number of motif occurrences in a region 200 bps around each of the peak summit as the evaluating criterion. Higher number indicates more accurate estimation of the peak summit locations. **A.** NRSF ChIP-Seq data. **B.** STAT1 ChIP-Seq data.

A.

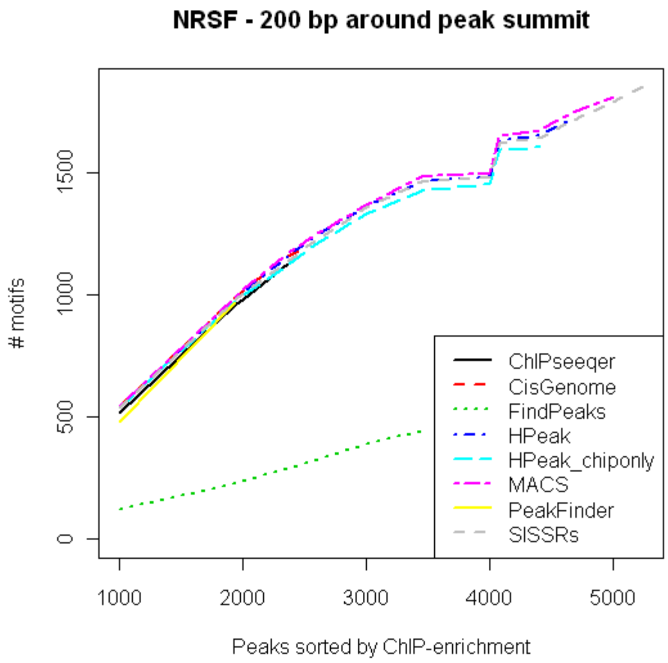

B.

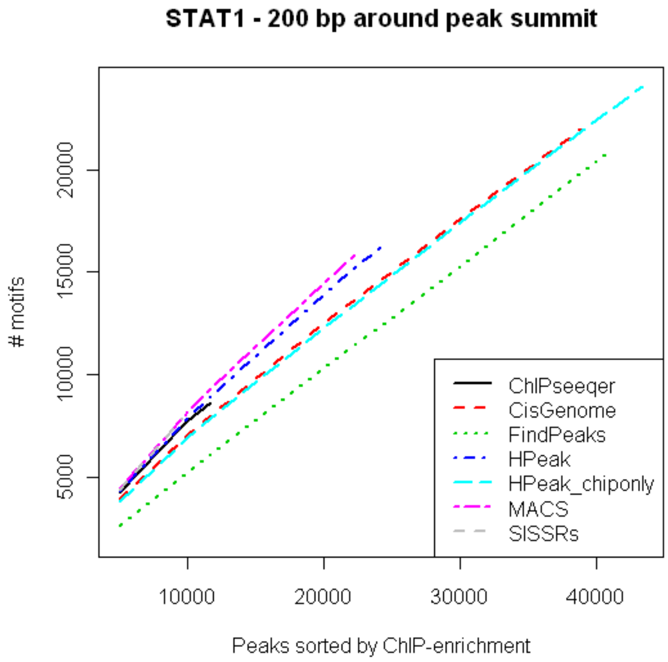

Figure S3. Histograms of peak lengths in four different ChIP-Seq datasets.

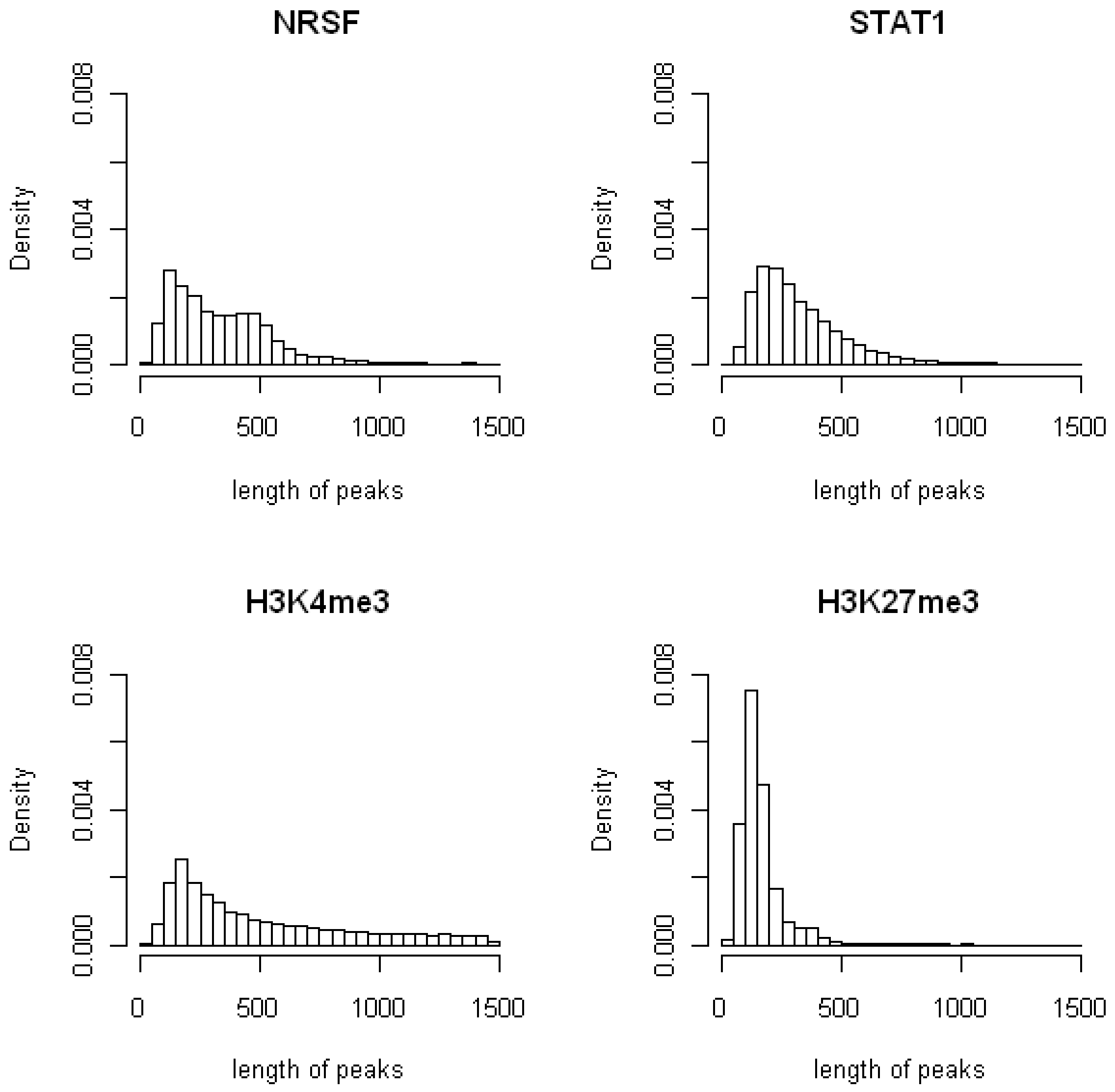

Figure S4. Comparison of model fitting using various distributions (Generalized Poisson (GP), Negative Binomial (NB) and Poisson distribution) to fit observed HDF count data on ChIP-enriched regions on Chromosome 1 of the STAT1 stimulated ChIP-Seq data. Curves shown are cumulative distribution functions (cdfs).

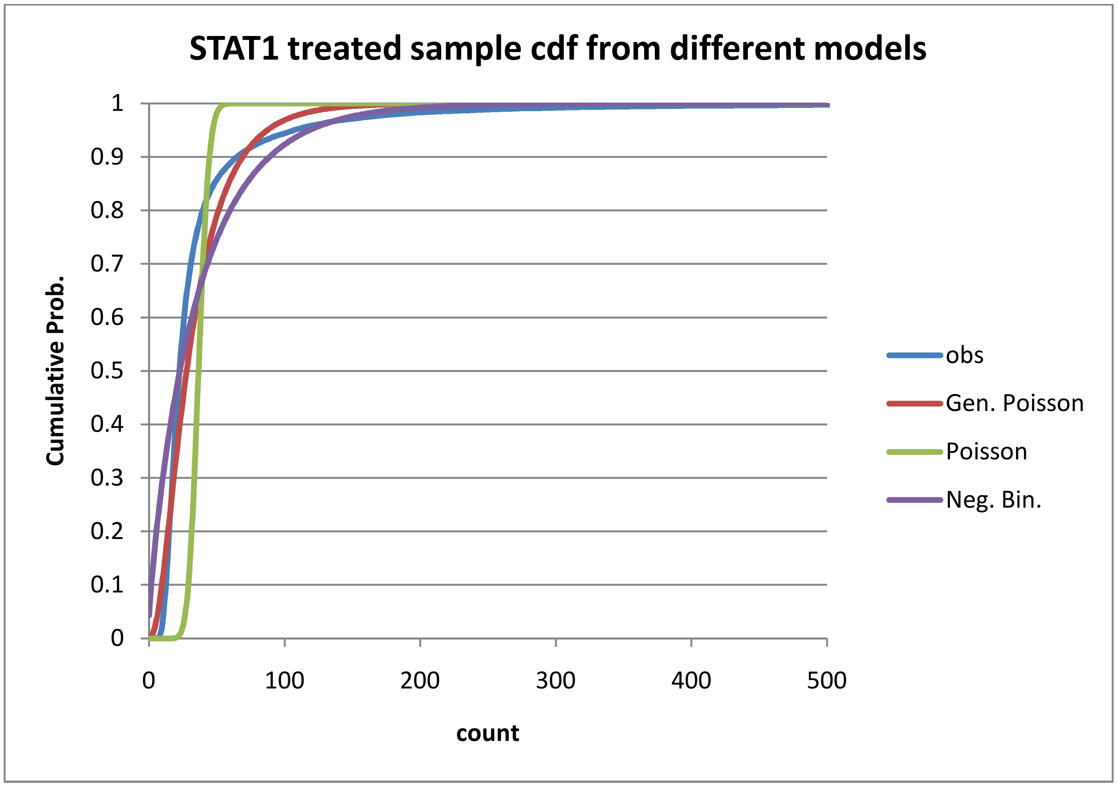

Figure S5. A. Illustration of the Hidden Markov model scheme. **B.** Illustration of the weighting scheme to account for uncertainties in the length of hypothetical DNA fragments.

A.

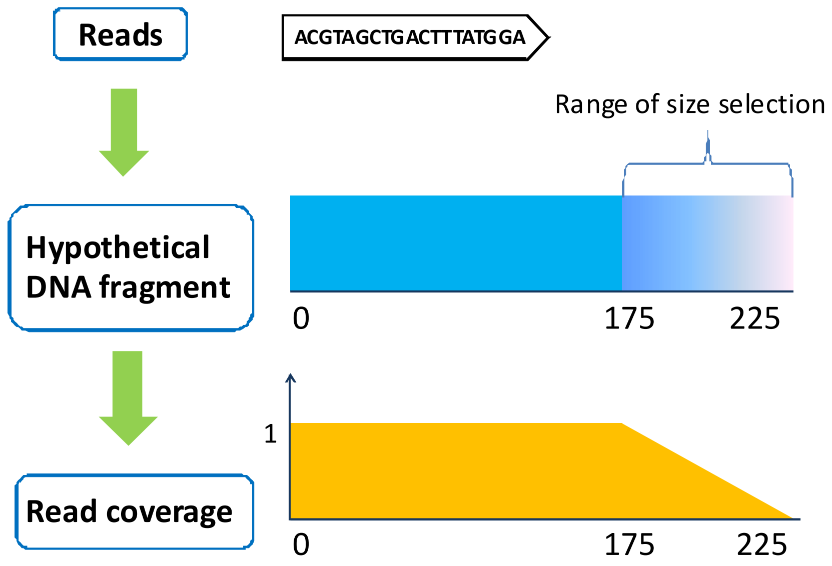

B.

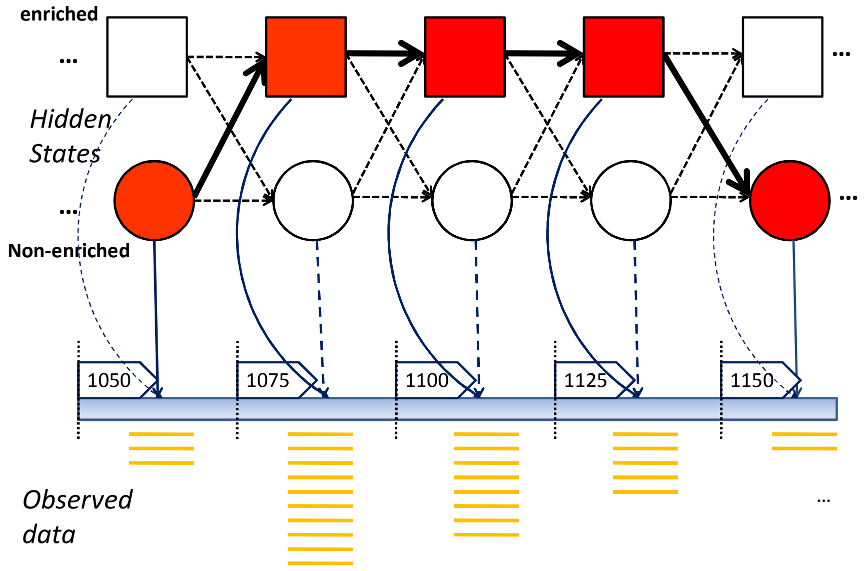

Supplement: Additional file 1 — More detailed information on methods and additional Tables and Figures. [file 1471-2105-11-369-S1.PDF]
